# Relationship of Gender and Smoking History in the Development of Hypothyroidism From Exposure to External Beam Radiation for Head and Neck Cancers

**DOI:** 10.14740/wjon835w

**Published:** 2014-06-25

**Authors:** Khurram Tariq, Fauzia Rana, Robert Zaiden, Lara Zuberi, Arezo Farhangi, Saif Ibrahim, Wajiha Tariq, Carlos Palacio, Farah Al-Saffar, Dat Pham

**Affiliations:** a Division of Hematology and Medical Oncology, University of Florida, Jacksonville, FL, USA

**Keywords:** Hypothyroidism, Head and neck cancer, External beam radiation

## Abstract

**Background:**

Patients with head and neck cancers (HNCs) are often treated with external beam radiation therapy (XRT). Unfortunately this therapy is not without its unintended consequences. One of these side effects includes the development of radiation-induced hypothyroidism. Our study is designed to pay special attention to variables like gender and smoking history and analyze their relationship with the development of hypothyroidism.

**Methods:**

Patients’ charts were reviewed over a period of 13 years from January 1, 2000 to November 30, 2013 to gather information on patients who had previously received XRTs for HNCs. We used the Tumor Registry Database at the University of Florida, College of Medicine in Jacksonville for this retrospective review. Patient characteristics were examined including age, gender, race/ethnicity and smoking history. Special attention was paid to the development of hypothyroidism (thyroid stimulating hormones > 5.0 with appropriate decrease in free serum T4) levels after exposure to XRT for HNCs. Results were then analyzed using the univariate statistical analysis which was done using the SAS software using a 0.05 alpha level of significance expressed in terms of odds ratio (OR) with 95% confidence intervals (CIs).

**Results:**

We found a total of 1,116 patients in our database who have received XRTs for HNCs. Out of these 72 (6.45%) patients developed hypothyroidism. Out of 263 African American patients 20 (7.6%) had hypothyroidism, whereas 49 (6%) out of 819 white people had this outcome. As for gender 32 out of 341 females (9.4%) and 40 out of 775 males had hypothyroidism. Thirty-five out of 544 (6.4%) with no family history and 19 (2.4%) out of 205 (9.3%) with positive family history for cancer had the outcome too. Finally 43 (6.5%) out of 664 smokers and 17 (8.3%) out of 206 non-smokers were found to develop hypothyroid as well. Both groups were found to have homogenous average age at diagnosis. When the univariate analysis was conducted, the strongest predicting variable was gender as hypothyroid patients were 1.90 (95% CI 1.17 - 3.09) times more likely to be females, and these results were statistically significant with P value of 0.008. More patients tended to be African Americans with OR 1.21 (CI 0.822 - 1.78), had positive family history of cancer (negative family history had OR 0.67 (CI 0.38 - 1.21), and less likely to be smokers (OR 0.77 (0.43 - 1.38). However, race, family history of cancer and smoking history did not achieve any statistical significance as evident by the P values.

**Conclusion:**

In our patient population, females were more likely to develop radiation-induced hypothyroidism.

## Introduction

Thyroid gland is often irradiated as an unintended consequence of external beam radiation therapy (XRT) for head and neck cancers (HNCs) [1]. The cells of the thyroid gland have a very low proliferation rate and this renders them the beneficial effect of radiotherapy resistance compared to the highly proliferative cell in the gastrointestinal track and the mammary gland [2]. However, the thyroid gland is by no means completely protected from the adverse affects of XRT. Over the years several reports have established the advent of hypothyroidism after radiation therapy for the treatment of HNCs and the role of certain variables like age and gender is an area under intense investigation [3]. The current study aims to specifically analyze the relationship of variables such as gender and smoking history with the development of hypothyroidism from XRT for the treatment of HNCs.

## Method

Tumor Registry Database at the University of Florida, College of Medicine in Jacksonville was utilized for our retrospective study to gather information on patients who had previously received XRTs for HNCs. Patients’ charts were reviewed over a period of 13 years from January 1, 2000 to November 30, 2013. General characteristics were examined including age, gender, race/ethnicity and smoking history. Hypothyroidism was defined as thyroid stimulating hormones (TSH) value greater than 5.0 along with appropriate decrease in free serum thyroxine (T4) levels after exposure to XRT. Results were then analyzed using the univariate statistical analysis which was done using the SAS software. Alpha level of significance was set to 0.05 and associations were expressed in terms of odds ratio (OR) with 95% confidence intervals (CIs).

## Results

We found a total of 1,116 patients in our database who have received XRTs for HNCs. Out of these 72 (6.45%) patients developed hypothyroidism. Out of 263 African American patients 20 (7.6%) had hypothyroidism, whereas 49 (6%) out of 819 white people had this outcome. As for gender 32 out of 341 females (9.4%) and 40 out of 775 males had hypothyroidism. Thirty-five out of 544 (6.4%) with no family history and 19 (2.4%) out of 205 (9.3%) with positive family history for cancer had the outcome too. Finally 43 (6.5%) out of 664 smokers and 17 (8.3%) out of 206 non-smokers were found to develop hypothyroid as well. Both groups were found to have homogenous average age at diagnosis.

When the univariate analysis was conducted, the strongest predicting variable was gender as hypothyroid patients were 1.90 (95% CI 1.17 - 3.09) times more likely to be females, and these results were statistically significant with P value of 0.008. More patients tended to be African Americans with OR 1.21 (CI 0.822 - 1.78), had positive family history of cancer (negative family history had OR 0.67 (CI 0.38 - 1.21) and less likely to be smokers (OR 0.77 (0.43 - 1.38). However, race, family history of cancer and smoking history did not achieve any statistical significance as evident by the P values. These findings are elaborated in Table 1.

**Table 1:** Descriptive and Univariate Statistics for Possible Predictors of Hypothyroidism in Cancer After External Beam Radiation

Predictors	Hypothyroid		Total	% Positive	OR (CI)	P value
	Yes	No				
Race					1.29 (0.74 - 2.22)	0.349
Black	20	243	263	7.6	1.21 (0.82 - 1.78)	
White	49	770	819	6	0.934 (0.80 - 1.09)	
Gender					1.90 (1.17 - 3.09)	0.008*
Female	32	309	341	9.4	1.50 (1.14 - 1.98)	
Male	40	735	775	5.2	0.79 (0.64 - 0.97)	
Patient status					1.76 (1.09 - 2.86)	0.02*
Alive	42	462	504	8.3	1.32 (1.07 - 1.62)	
Dead	30	582	612	4.9	0.75 (0.57 - 0.99)	
Family history					0.67 (0.38 - 1.21)	0.181
No	35	509	544	6.4	0.89 (0.72 - 1.08)	
Yes	19	186	205	9.3	1.32 (0.89 - 1.93)	
Tobacco history					0.77 (0.43 - 1.38)	0.38
Current/previous	43	621	664	6.5		
Never used	17	189	206	8.3		
Mean age at diagnosis (SD)	60.93 (10.66)	59.59 (12.19)				0.363

## Discussion

XRT is an integral part of the treatment for HNCs. In early-stage tumors, the treatment may involve XRT alone; however, in more advanced stages the HNCs are treated with XRT along with surgery and/or chemotherapy [4]. The incidence of clinical hypothyroidism described in the literature is between 5% and 10% [5, 6]. Clinical hypothyroidism is defined as the increase in TSH above 5.0 and a decrease in free thyroxine levels (free T4) in the serum. Results obtained from our patient population are in agreement with these findings. In the current study 6.45% of our patient population developed clinical hypothyroidism. As expected, the incidence of hypothyroidism is much higher in the patients receiving radiation therapy in addition to surgical interventions involving the thyroid gland [1-6]. Our lower incidence of clinical hypothyroidism is in agreement with the published date deduced from patients treated with XRT alone.

Both the exact dose of XRT and the mechanism behind the destruction of thyroid gland are not well understood. There is conflicting evidence on the exact amount of XRT needed to affect the thyroid gland with a wide range from 3,000 to 8,000 rads [7, 8]. On the other hand, several mechanisms of injury to the thyroid have been proposed over the years ranging from vascular or immunologically medicated damage to prevention of cell division to the direct follicular destruction of the thyroid gland [4]. The most widely accepted mechanism involves the micro-and macrovascular damage to the thyroid gland itself along with the area around it which then leads to the ischemia and fibrosis leading to the destruction of thyroid gland’s synthetic ability [9].

**Figure 1:**
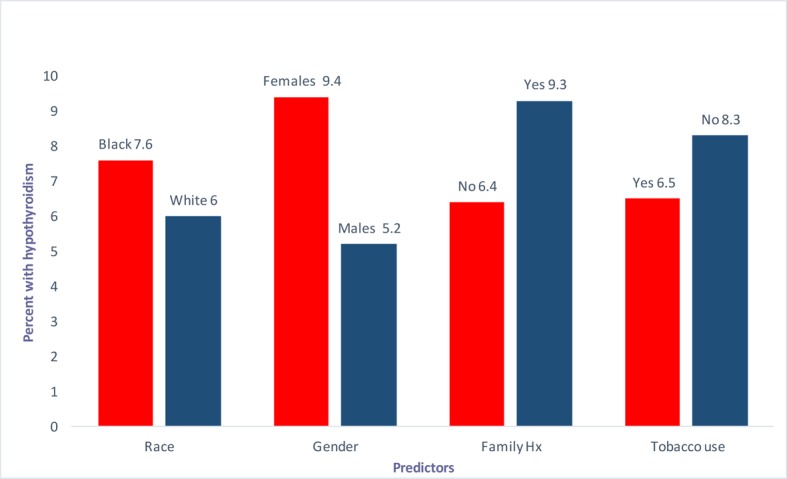
Predictors (race, gender, family history and tobaccos use) vs. percentage with hypothyroidism.

Certain risk factors have been consistently reported throughout the literature. These include the location of the tumor, the extent/intensity of the XRT and the treatment modality or modalities like chemotherapy or surgery used in conjunction with the radiation therapy [9]. Variables like age and gender have been studied previously but the research is very limited. Several authors including Mercado et al, Tell et al and Aich et al, have shown no correlation between gender and iatrogenic hypothyroidism secondary to XRT [4, 10, 11]. This makes our current study very unique in that we looked at gender at our institution and the advent of hypothyroidism over a 13-year period. A detailed version of our results is mentioned in Table 1. A bar graph (Fig. 1) is also shown below for a better visual depiction.

Interestingly we found that the female gender was positively associated with the development of iatrogenic hypothyroidism with an OR of 1.90 and a 95% CI 1.17 - 3.09. Using a univariate analysis we were also able to show that the difference between the genders was statistically significant with a P value of 0.008. Gender predilection for iatrogenic hypothyroidism can have significant implications on public awareness programs and on future guidelines for more stringent follow-up of the TSH and free T4 in the female gender.

Another area of controversy is the effect of cigarette smoking on thyroid dysfunction. Published research on the influence of cigarette smoking on thyroid malfunction has shown both hypothyroidism and hyperthyroidism. Yet in other studies smoking has shown to have no effect on thyroid function [12-18]. Impact of smoking thus remains an area of intense investigation and is a variable that was studied in the current study as well. We found that smoking was negatively associated with iatrogenic hypothyroidism with an OR of 0.77 and a CI 0.43 - 1.38. While on the surface there appears to be a protective effect of cigarette smoking, this finding did not bode statistical significance with a P value of 0.38.

Our study reports some very significant findings; however, it also emphasizes the need to carry out future investigations based on data pooled from multiple cancer centers and possible future meta-analysis of predictors of post-radiotherapy hypothyroidism. This can help provide more information about other possible risk factors and more in-depth analysis of the aforementioned ones. Other interesting variables to examine can be gender differences in family history of hypothyroidism, presence of thyroid and non-thyroid autoimmune diseases as well as the effect of preceding, concomitant, and subsequent chemotherapy in patients based on gender.

### Conclusion

In our patient population, the development of hypothyroidism after XRT for the treatment of HNCs was more common among the female population. There is also some evidence that smoking may have a protective effect on the development of hypothyroidism; however, this association could not reach statistical significance. More patients tended to be African Americans, with positive family history of cancer, and less likely to be smokers. However, these variables did not achieve any statistical significance emphasizing the need for future research involving collaboration between multiple centers on a much bigger scale. Bigger sample size will allow more accurate estimation of the possible protective effect and its magnitude. It will also enable us to better estimate possible statistical significance. These findings can have future public policy implications for guidelines pertaining to more stringent screening of radiation-induced hypothyroidism in the subgroups at the highest risk.
